# Association between asymptomatic submicroscopic and microscopic malaria infections and anemia: A study in southern Benin

**DOI:** 10.1371/journal.pone.0317345

**Published:** 2025-01-24

**Authors:** Alejandro Rojas Chaves, Yannelle Dossou, Armel Djènontin, Elisée Adimi, Romuald Akoho, Justine Bailly, Aziz Bouraïma, Déborah Matondo, Yolande Sissinto, Dismand Houinato, Achille Massougbodji, Célia Dechavanne, Gilles Cottrell

**Affiliations:** 1 Université Paris Cité, IRD, MERIT, F-75006, Paris, France; 2 Institut de Recherche Clinique du Bénin/IRCB, Abomey-calavi, Bénin; 3 Centre de Recherche Entomologique de Cotonou (CREC), Cotonou (Bénin) and Centre de Recherche Pour la Lutte Contre les Maladies Infectieuses Tropicales (CReMIT), Université d’Abomey-Calavi (UAC), Cotonou, Bénin; 4 Centre d’Etude et de Recherche sur le Paludisme Associé à la Grossesse et à l’Enfance (CERPAGE), Faculté des Sciences de la Santé, Cotonou, Bénin; 5 Faculté des Sciences de la Santé, Université d’Abomey Calavi, Cotonou, Bénin; Para Federal University, BRAZIL

## Abstract

**Introduction:**

Recently, efforts to eliminate malaria have shifted focus from symptomatic cases alone to include asymptomatic carriers, who are now recognized as significant contributors to the disease’s transmission and control. This study examines the relationship between asymptomatic malaria infection and hemoglobin levels in Benin.

**Methods:**

A cohort in Benin was enrolled and categorized into three age groups (under 5 years, 5–15 years, and over 15 years) for follow-up from August to November 2021. Participants were monitored over two months and assessed for malaria infection through microscopy and polymerase chain reaction (PCR) during their three visits. A questionnaire was employed to gather general and clinical characteristics. Multivariate models were utilized to analyze the associations between asymptomatic infection, anemia, and hemoglobin levels.

**Results:**

Among 393 participants, 58.2% were diagnosed at enrolment with malaria via PCR, and 30.5% through microscopy. Anemia, defined as hemoglobin levels < 11 g/dL, was observed in 40.5% of the cohort. The risk of anemia declined with increasing age (OR for ages 5–15: 0.64, 95% CI [0.37; 1.09]; OR for over 15 years: 0.34, 95% CI [0.20; 0.59]) compared to children under 5. Individuals with both microscopic and submicroscopic infections exhibited higher odds of anemia (respectively OR = 4.15, 95% CI [2.41; 7.13] and OR = 2.09 [1.22; 3.57]) relative to those uninfected. Hemoglobin levels were consistently lower in participants with microscopic malaria across all age groups (β = -2.73, 95% CI [-3.41; -2.05] for those under 5, β = -1.35, 95% CI [-1.89; -0.82] for ages 5–15, and β = -0.72, 95% CI [-1.34; -0.07]) compared to non-infected individuals.

**Discussion:**

Our findings suggest that asymptomatic malaria infections, including submicroscopic cases, are associated with anemia and decreased hemoglobin levels. This underscores the importance of employing ultrasensitive diagnostic methods for such infections and acknowledging their potential health implications.

## Introduction

After a steady improvement between 2000 and 2015 in the global fight against malaria, the progress has stalled in recent years [1]. Some of the reasons include lack of access to prevention methods and treatment, inappropriate access to health services, drug resistance and the impact of the COVID-19 pandemic [2,3]. According to the World Health Organisation (WHO), in 2020 there were 241 million of cases reported, which represents almost a 10% increase with respect to 2019.

The incidence of malaria increased from 56 to 59 cases per 1,000 people, and the number of malaria-related deaths rose by 12% compared to 2019, totaling 627,000 deaths in 2020 [1]. Africa remains the most affected region, accounting for 95% of all cases and 96% of all global malaria deaths, with *Plasmodium falciparum* responsible for approximately 95% of infections in 2020 [1,4,5]. In Benin, both cases and mortality have been on the rise since 2016, reaching up to 2,516,465 cases and 2,336 deaths in 2020 [1,6]. Although there have been improvements in malaria control methods in Benin, challenges such as vector resistance to insecticides persist [1,6]. Until recently, efforts to eradicate malaria primarily targeted symptomatic cases, but there is now recognition of the significant role that asymptomatic carriers play in the transmission and control of the disease [1]. Despite a lack of standardized definition, asymptomatic malaria is generally defined as the detection of asexual or sexual forms of the parasite in the absence of fever [7]. Other criteria might include self-reported fever or antimalarial treatment use [2,8]. A major challenge in identifying such cases is the typically lower parasitemia levels in asymptomatic infections compared to symptomatic ones. Some asymptomatic infections are even submicroscopic, making them undetectable by traditional methods like microscopy or rapid diagnostic tests (which have a limit of detection around 100,000 parasites per mL) but identifiable via more sensitive molecular methods such as Polymerase Chain Reaction (PCR), with a detection limit of around 1 parasite per μL [2,8]. The proportion of submicroscopic infections varies by epidemiological context, with high transmission areas potentially having a minimum of 20% of infections being submicroscopic, while in low transmission areas this can reach 70–80% [8]. Asymptomatic infections constitute the majority of the malaria reservoir and contribute most to transmission [2,4,9–11]. However, their prevalence is often underestimated because commonly used diagnostic methods like microscopy and rapid tests lack sensitivity for low parasitemia detections [1,8]. Asymptomatic infections are significant concerning health outcomes [2,8], though the knowledge about these impacts remains limited. Some studies suggest potential effects on anemia, T cell downregulation, increased risk of secondary bacterial infections like non-typhoidal salmonella, and impaired cognitive function affecting school performance [7,12–14]. In pregnant women, asymptomatic infections in high transmission areas often result in placental malaria, which is linked to preterm births, low birth weights, and peripartum hemorrhage [11,13]. Anemia may result from chronic low-grade hemolysis coupled with symptomatic recurrent episodes that can lead to splenomegaly, depending on the number of episodes [7,12]. The objective of this work is to investigate the consequences of both microscopic and submicroscopic asymptomatic malaria infections on anemia and hemoglobin levels across different age groups within a rural community in southern Benin, adjusted for potential risk factors.

## Methods

### Enrolment and follow-up

**Fig 1:**
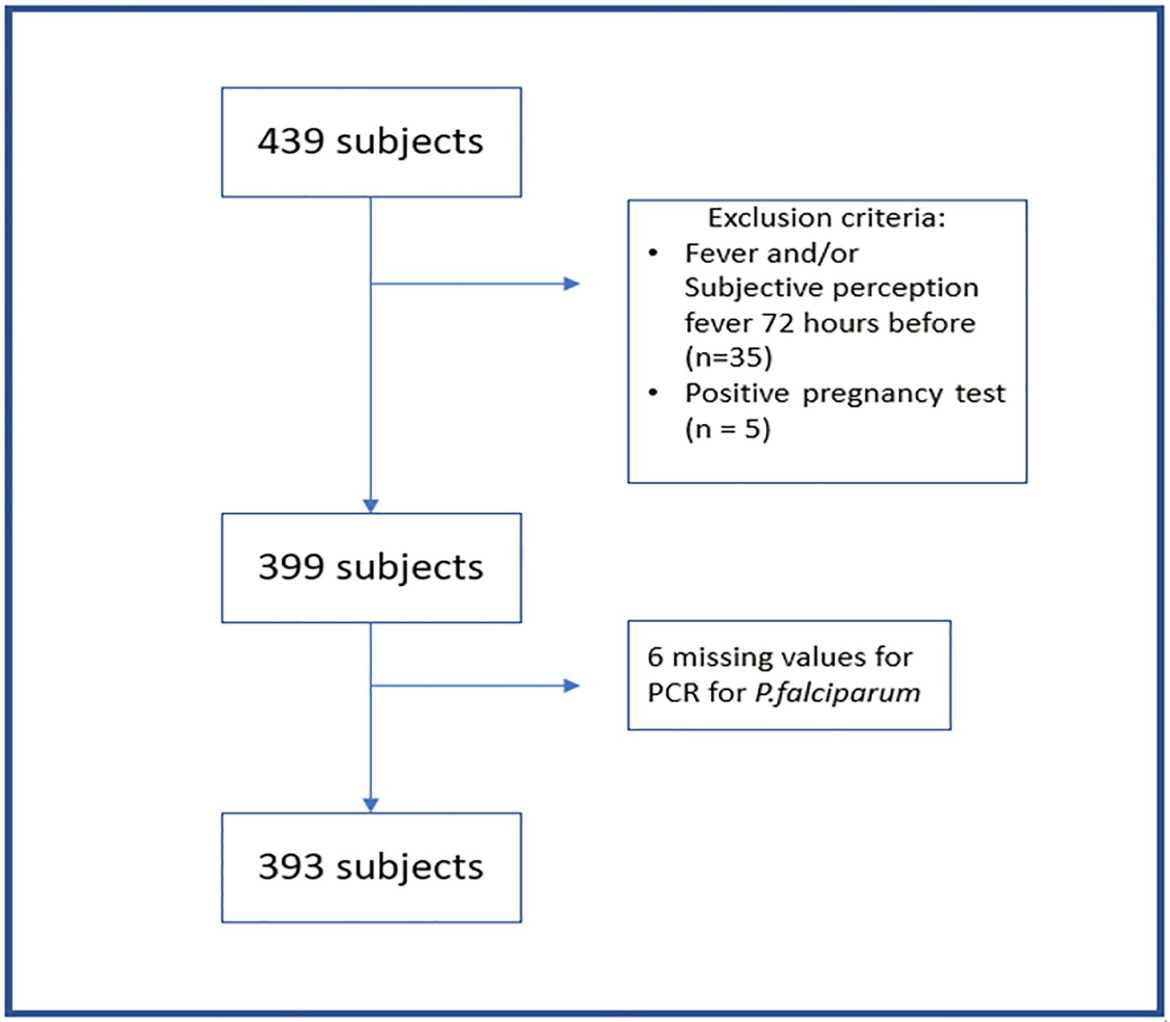
Flowchart of the study population.

Data were collected between August and November 2021 in southern Benin at enrolment of a longitudinal pilot study aimed at examining the dynamics of asymptomatic malaria infection. Healthcare workers collected the data using smartphones to ensure its completeness and quality. The study sample included 439 asymptomatic individuals, grouped into three distinct age categories: under 5 years old, between 5 and 15 years old, and over 15 years old. Subjects who had a fever (defined as a body temperature above 37.5 °C at the time of examination) or reported having a fever within the past 72 hours, those planning to travel over the next 3 months, and women with a positive urinary pregnancy test (Micropoint Rapid Diagnostic Test for detecting the Chorionic Gonadotropic Hormone) were treated according to national guidelines and excluded from follow-up. After excluding additional subjects without conclusive PCR results for P. falciparum infection, 393 participants remained for analysis (see Fig 1). Additionally, for each observation, both thick smears and PCR tests were performed, and hemoglobin levels were measured.

### Malaria diagnosis

#### Diagnosis of Plasmodium infection by RDT

RDTs (Rapid Diagnostic Tests) were conducted at the time of inclusion or during follow-up in case of fever, utilizing the detection of the PfHRP2 antigen (Histidine Rich Protein 2) [15].

#### Diagnosis of Plasmodium infection by thick blood smears

The Lambaréné method involved using 10 μL of whole blood, evenly spread over an area of 10x18 mm, which was then dried and stained with 10% Giemsa. Thick blood smears were deemed positive if at least one trophozoite was observed after examining 1,000 leukocytes. The different species of *Plasmodium* were identified by microscopy and PCR [16].

#### Diagnosis of Plasmodium species infection by PCR

Whole blood samples (200 μL) were stored at -20°C until use. DNA was extracted using the QIAamp DNA Mini Kit (Qiagen, Hilden, Germany) according to the manufacturer’s instructions, and the extracted DNA was stored at -20°C until further analysis. The primers used for the EvaGreen assay targeted three independent Plasmodium genes: *P*. *falciparum* cytochrome b mitochondrial (Pf cytb), P. malariae 18S rRNA sequence (Pm 18s), and *P*. *ovale* dihydrofolate reductase (Po dhfr) genes (as detailed in S1 Table). The qPCR assays were conducted on the Viia7^™^ Real-Time PCR System (Thermo Fisher Scientific, Massachusetts, US) and QuantStudio 5 (Applied Biosystems, Massachusetts, US). The thermal profile for the qPCR was as follows: an initial step at 95°C for 15 minutes, followed by 40 cycles of 15 seconds at 95°C, 20 seconds at 63°C for Pf and Pm, or 58°C for Po, and 20 seconds at 72°C. Each reaction well for the qPCR assays of *P*. *falciparum*, *P*. *malariae*, and *P*. *ovale* contained 3 μL of 5X HOT FIREPol EvaGreen Mix Plus (Solis BioDyne, Tartu, Estonia), 0.5 μM of each primer (Pfcytb, Pm18s, and Podhfr), and 2.5 μL of DNA, making a total reaction volume of 15 μL. Primers were supplied by Integrated DNA Technologies.

#### Anemia

Hemoglobin levels were measured using the HemoCue^®^ Hb 201+ System. A single drop of peripheral blood was collected via a finger prick to fill a HemoCue^®^ microcuvette. Anemia was defined as a binary outcome, with levels below 11 g/dL considered indicative of anemia [12]. Hemoglobin levels were the primary outcomes measured.

#### Clinical groups

Asymptomatic malaria infection was classified as a categorical variable with three levels based on PCR and microscopy results:

- Microscopic infection: positive microscopy test
- Submicroscopic infection: positive PCR test and negative microscopy test
- No infection: both tests negative

### Statistical analysis

The variables selected for this analysis included demographic characteristics such as age, sex, educational level, professional status, and number of household members. Additionally, the use of preventive measures against malaria, including long-lasting insecticidal nets (LLINs), sprays, or mosquito coils, behaviors related to LLIN use, the use of antimalarial treatments or consumption of herbal tea, and temperature at the time of examination were considered. All statistical analyses were conducted using R Statistical Software (v2.2), utilizing the packages epiDisplay and dplyr [17–19]. The relationships between hemoglobin levels (a quantitative variable) and anemia (a binary variable) with the covariates were analyzed using linear and logistic regression, respectively. Variables that showed a p-value less than 0.20, or that had an effect size deemed important in univariate regression models, were included in the multivariate analysis, apart from asymptomatic malaria infection and age group, which were included in all multivariate models. A stratified analysis by age group was also conducted for both outcomes, with the significance level set at 0.05. When comparing nested logistic regression models, the Akaike Information Criterion (AIC) was used to select the best-fitting model. For linear regression, models were compared using the adjusted R^2^.

### Ethical consideration

This study received ethical approval from the institutional ethics committee at the Centre de Recherche Entomologique de Cotonou (CREC).

## Results

**Fig 2:**
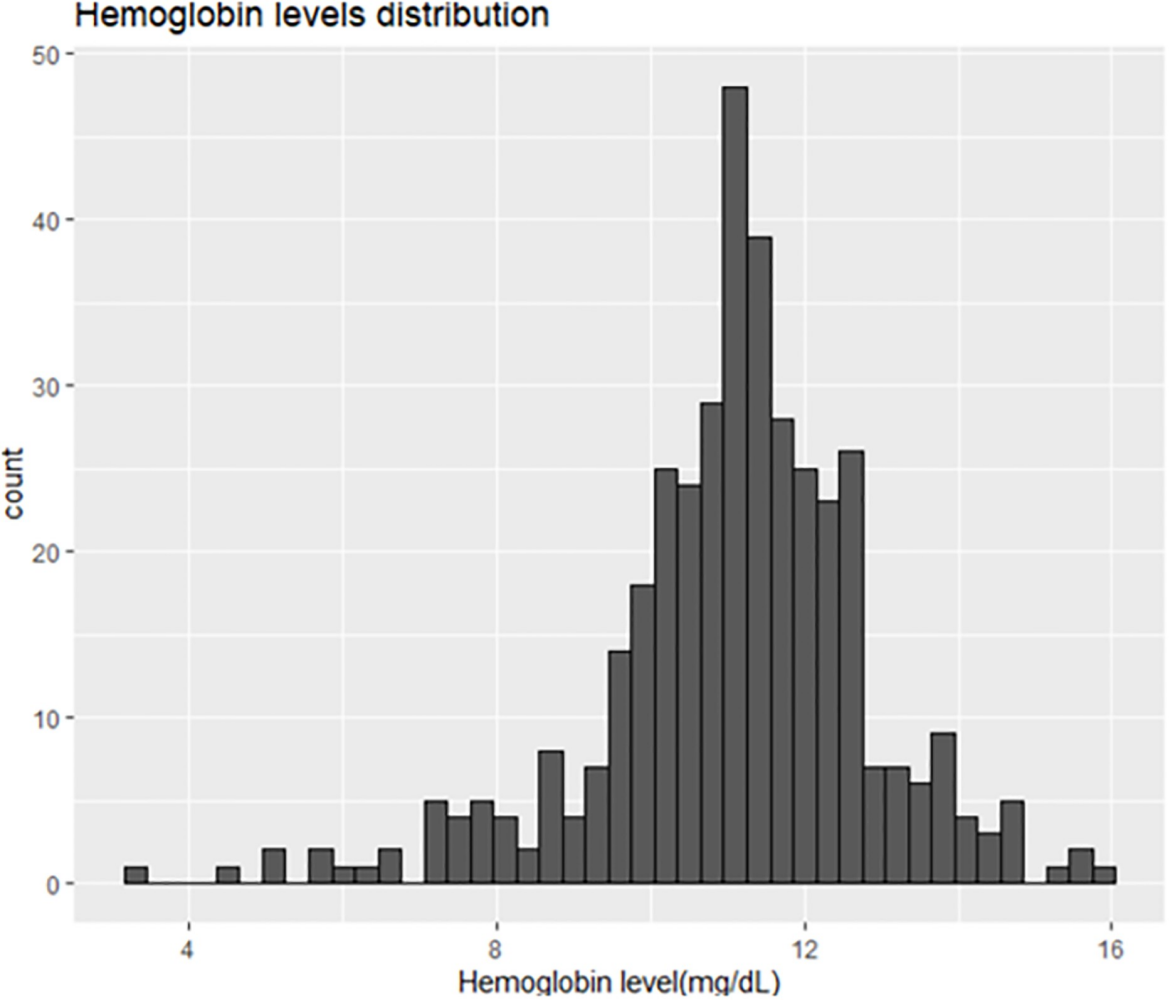
Hemoglobin level distribution of included participants.

Out of the 439 subjects included in this study, 35 (8.0%) had a fever recorded at the examination point or a history of fever in the previous 72 hours. Their mean hemoglobin level was 11.07 g/dL; 19 (54.3%) had a microscopic infection, and 7 (20.0%) had a submicroscopic infection. Five women tested positive for pregnancy; none had a fever nor reported experiencing fever in preceding days. In total, 393 subjects were included in the analysis, with their characteristics outlined in Table 1. The overall proportion of anemia was 40.5% (n = 159), and more than half of the anemic individuals were female (55.2%). Across all age groups, most individuals did not have anemia, with anemia prevalence ranging from 27.5% in those older than 15 years to around 47% in the other two age groups. Most participants reported using long-lasting insecticidal nets (LLIN), with 85.0% coverage, and 25.7% supplemented LLIN use with other preventive measures. Only six participants (1.5%) did not utilize any malaria prevention methods. Positive blood smears were found in 120 participants (30.5%), while 228 (58.0%) showed positive results in PCR tests. Among those with positive PCR results (228/393), 213 were infected with *P*. *falciparum*, 12 were co-infected with *P*. *falciparum* and *P*. *malariae*, and 2 with *P*. *malariae* and *P*. *ovale*. Only one individual was infected with *P*. *falciparum*, *P*. *malariae*, and *P*. *ovale* simultaneously. In the study population, 41.7% (164 subjects) tested negative for malaria, while 109 (27.7%) were classified with submicroscopic infection, and 120 (30.5%) had microscopic infection. Regarding parasite distribution, among microscopic infections, *P*. *falciparum* was detected in 118 samples (52.21%) and in 108 submicroscopic infections (47.79%). *P*. *malariae* was found in 12 microscopic infections and 3 submicroscopic infections. *P*. *ovale* was found only in 2 microscopic and 1 submicroscopic infections. Notably, *P*. *malariae* and *P*. *ovale* infections were part of combined infections. Interestingly, most subjects with microscopic infection had anemia (57.5%). Regarding parasitemia, those with anemia exhibited higher parasitemia (1.39, IQR [0–454]) compared to those without anemia. Hemoglobin levels were distributed as shown in Fig 2, with a mean of 11.01 g/dL and a standard deviation of 1.78. Among participants with anemia, 135 (84.91%) had mild anemia (8 g/dL < Hb < 11 g/dL), 22 (13.83%) had moderate anemia (5 g/dL < Hb ≤ 8 g/dL), and only 2 (1.26%) had severe anemia (≤ 5 g/dL).

**Table 1:** Characteristics of participants with an asymptomatic malaria infection: Overall and by anemia status (n = 393).

Variable		Overall	Anemia n (%)^1^		p.value^2^
		N (%)	Yes (n = 159)	No (n = 234)	
Sex					
	Female	217 (55.22)	85 (53.46)	132 (56.41)	0.606
	Male	176 (44.78)	74 (46.54)	102 (43.59)	
Age group					
	Less than 5 years	128 (32.57)	60 (37.74)	68 (29.06)	<0.001
	5–15 years	134 (34.10)	63 (39.62)	71 (30.34)	
	More than 15 years	131 (33.33)	36 (22.64)	95 (40.60)	
Use of bednet night before					
	Yes	292 (74.30)	118 (74.21)	174 (74.36)	0.480
	No	42 (10.63)	20 (12.58)	22 (9.40)	
	Unknown	59 (15.07)	21 (13.21)	38 (16.24)	
Other measures					
	Mosquito coil	135 (34.35)	57 (35.85)	78 (33.33)	0.382
	Sprays	4 (1.02)	0 (0.00)	4 (1.71)	
	None	178 (45.29)	69 (43.40)	109 (46.58)	
	Unknown	76 (19.34)	33 (20.75)	43 (18.38)	
Consumption of tisane^3^					
	Yes	51 (12.98)	14 (8.81)	37 (15.81)	0.047
	No	342 (87.02)	145 (91.20)	197 (84.19)	
Use of antimalarial treatment					
	Yes	22 (5.60)	14 (8.81)	8 (3.42)	0.026
	No	371 (94.40)	145 (91.19)	226 (96.58)	
Number of household members					
	5 or less	165 (41.98)	63 (39.62)	102 (43.59)	0.006
	6–10	211 (53.69)	85 (53.46)	126 (53.85)	
	More than 10	17 (4.33)	11 (6.92)	6 (2.56)	
Malaria Infection					
	No infection	164 (41.73)	45 (28.30)	119 (50.85)	<0.001
	Submicroscopica	109 (27.74)	45 (28.30)	64 (27.35)	
	Microscopic^a^	120 (30.53)	69 (43.40)	51 (21.80)	
Parasitemia (parasite/ μL)					<0.001
	Mean (SD)	7833 (43964)	7870 (52115)	7807 (37551)	
	Median (IQR)	1.1 (0–328)	1.39 (0–454)	1.13 (0–281)	

^1^Threshold: 11 g/dL.

^2^Fisher exact.test.

^3^Refers to a regular consumption, several times per week of herbal tea.

^3^Submicroscopic infection defined as negative thick film and positive PCR, while for microscopic infection, thick film is positive.

### Multivariate analysis

Variables included for multivariate analysis were malaria infection, hemoglobin levels or anemia, sex, age group, and use of antimalarial drugs.

In the final logistic model, anemia was adjusted for malaria infection, age group, and prior use of antimalarial drugs. Within the overall sample, the risk of anemia was higher in the presence of any type of infection compared to no infection, with the odds being significantly higher in cases of microscopic infection, where the odds ratio was 4.15 (95% CI [2.41; 7.13]). Additionally, in this model, the risk of developing anemia decreased with increasing age. As depicted in Table 2, the odds ratio of having anemia was higher for microscopic infections across all three age groups compared to submicroscopic infections, and this odds ratio decreased with age.

**Table 2:** Factors associated with anemia stratified by age group: By logistic regression.

Variable	Overall model		Less than 5 years		5–15 years		More 15 years	
	OR (95%CI)	p.value	OR (95%CI)	p.value	OR (95%CI)	p.value	OR (95%CI)	p.value
**Infection classification**								
*(ref*: *No infection)* **Submicroscopic**	2.09 (1.22; 3.57)	<0.001	1.59 (0.68; 3.69)	0.284	3.99 (1.37; 11.62)	0.011	1.47 (0.58; 3.75)	0.419
**Microscopic**	4.15 (2.41; 7.13)	<0.001	8.51 (2.61; 27.72)	<0.001	7.17 (2.68; 19.23)	<0.001	1.76 (0.68; 4.52)	0.242
**Age group** *(ref*: *<5 years old)*								
**5–15 years old**	0.64 (0.37; 1.09)	0.101	-		-		-	
**>15 years old**	0.34 (0.20; 0.59)	<0.001						
**Antimalarial drugs**	2.36 (0.92; 6.09)	0.069	1.28 (0.24; 6.74)	0.772	3.88 (0.92; 16.39)	0.065	1.46 (0.12; 17.20)	0.765
*(ref*: *No use)*								
**AIC**	496.27		168.48		170.75		160.52	

The final multivariate linear model, which utilized hemoglobin as a quantitative variable, was adjusted for age group, sex, and malaria infection status. As shown in Table 3, individuals with microscopic infections had the lowest hemoglobin levels, with an average hemoglobin level 1.44 g/dL lower than that of non-infected participants. Those with submicroscopic infections had an average hemoglobin level 0.75 g/dL lower than the malaria-negative group. When the analysis was stratified by age group (Table 3), a decreasing impact of microscopic infection with increasing age was observed. This trend was not evident for submicroscopic infections, which continued to have an impact on anemia in the 5–15 year and over 15-year age groups. In the over 15 years age group, females had a substantially lower mean hemoglobin level than males.

**Table 3:** Factors associated with the hemoglobin level stratified by age group: Linear regression.

Variable	Overall model		Less than 5 years		5–15 years		More 15 years	
	Coefficient (95%CI)	p.value	B (95%CI)	p.value	B (95%CI)	p.value	B (95%CI)	p.value
**Intercept**	11.28 (10.93; 11.64)	<0.001	11.11 (10.68–11.54)	<0.001	11.48 (10.98; 11.99)	<0.001	13.61 (12.96; 14.25)	<0.001
**Infection classification**								
*(ref*: *No infection)* **Submicroscopic**	-0.75 (-1.14; -0.36)	<0.001	-0.38 (-0.99;0.23)	0.220	-0.95 (-1.54; -0.36)	0.002	0.63 (-1.40; -0.04)	0.036
**Microscopic**	-1.44 (-1.82; -1.05)	<0.001	-2.73 (-3.41; -2.05)	<0.001	-1.35 (-1.89; -0.82)	<0.001	0.72 (-1.34; 0.07)	0.076
**Age group** *(ref*: *<5 years old)*								
**5–15 years old**	0.64 (0.24; 1.03)	0.002	-		-		-	
**>15 years old**	1.61 (1.22; 2.00)	<0.001						
**Sex** *(ref*: *Males)*	-0.58 (-0.90; -0.25)	<0.001	-0.04 (-0.48; 0.56)	0.88	-0.35 (-0.09; 0.79)	0.118	-1.98 (-2.59; -1.37)	0.765
**Adjusted R** ^**2**^	21.95%		32.79%		18.96%		23.45%	

## Discussion

Asymptomatic malaria is highly prevalent across all age groups in southern Benin, ranging from about 40% in children under 5 years old to approximately 70% in school-aged children (5–15 years old). The prevalence detected by PCR was significantly higher in all age groups compared to estimates from microscopy, illustrating how recommended diagnostic methods underestimate asymptomatic cases. This finding aligns with studies from Kenya and Tanzania, among others [12,20,21]. Both microscopic and submicroscopic asymptomatic infections were linked to an elevated risk of anemia. Subjects with microscopic infections had the highest risk (OR = 4.15, 95% CI [2.41; 7.13]), while those with submicroscopic infections also showed a notably increased risk (OR = 2.09, 95% CI [1.22; 3.57]) compared to uninfected individuals. The malaria-related risk of anemia decreased with age, a trend similarly observed in hemoglobin levels, where microscopic infections were associated with a greater reduction compared to submicroscopic infections across all age groups. This impact also lessened with age. Supported by studies from Indonesia, microscopic infections were found to reduce hemoglobin concentration more than submicroscopic and no infections [22,23]. Similarly, a study from Cameroon identified high parasitemia and female sex as risk factors for anemia development [24]. In line with our findings, a study in Uganda showed higher microscopic parasitemia prevalence in children compared to adults, albeit inclusive of symptomatic participants [25]. According to a prevalence study in Tanzania, asymptomatic infections elevate anemia risk, with odds ratios decreasing across age groups utilizing both PCR and microscopy tests [21]. Another study from Indonesia found age to be a risk factor for anemia in those infected with *P*. *falciparum* but not *P*. *vivax* [14]. Anemia risk was higher among asymptomatic individuals with submicroscopic infections. A Tanzanian study highlighted an increased anemia prevalence in children aged 6–9 compared to those aged 10–13 in the context of malaria infection [26]. These findings, including our own, suggest that as age increases, the development of immunity against malaria likely contributes [11,27]. Adults are more adept at limiting parasitemia, which may account for the higher prevalence of submicroscopic malaria within this group [28]. The inverse relationship between hemoglobin levels and parasite density is consistent with previous studies [29]. However, varying definitions of anemia should be noted when comparing literature, as different thresholds may be used. Sex differences were evident in hemoglobin levels, with females showing higher odds of anemia, particularly those older than 15. These differences may stem from cultural practices and activities affecting vector exposure. A review suggests that females may clear parasites faster due to hormonal influences and antibodies, possibly explaining lower malaria prevalence among females, though further research is needed [30]. The primary strength of this study was the use of both PCR and microscopy, enabling the distinction between submicroscopic and microscopic infections, and differentiating symptomatic from asymptomatic carriers, thereby focusing on a less-documented group. Limitations include relying solely on enrollment data, thus not establishing causal relationships or capturing all confounders, such as sickle cell, and not tracking if asymptomatic episodes became symptomatic later [13]. The study also lacked adjustments for certain confounders. Anemia’s multifactorial nature requires consideration of alternate causes, such as genetics, nutrition, parity, and infections like hookworm and schistosomiasis [19,22,31–34]. Beyond biological factors, variations in malaria prevalence and anemia may also result from socioeconomic, dietary, or environmental differences [32]. The lack of standardized criteria for defining asymptomatic malaria complicates comparisons, with calls in the literature for a more precise definition that accounts for other malaria symptoms or previous treatment [2,8,35].

## Conclusion

Anemia is a multifactorial issue that poses a significant public health concern, and an association with asymptomatic malaria was identified in a highly prevalent region of southern Benin. The impact of the infection varied across different infection types and age groups, with children under 5 years old experiencing the most significant reduction in hemoglobin levels. It is essential to implement access to ultrasensitive methods and tailored interventions to facilitate case detection and prevent serious outcomes.

## Supporting information

S1 Table Primers used for the Plasmodium detection by PCR. (DOCX)
