# Supplementary material for: Association between asymptomatic submicroscopic and microscopic malaria infections and anemia: A study in southern Benin
Source: PLoS One. 2025 Jan 24;20(1):e0317345. doi: 10.1371/journal.pone.0317345 (PMC11759402; doi:10.1371/journal.pone.0317345)
Supplement: S1 Table — (DOCX) [file pone.0317345.s001.docx]

**Supplementary Table 1:** Primers used for the *Plasmodium* detection by PCR.

| **Species** | **Oligo name** | **Target region** | **Oligo final concentrations (μM)** | **Sequence (5’-3’)** | **Supplier** |
| --- | --- | --- | --- | --- | --- |
| *P. falciparum* | *Pf*cytb_m_F2 | cytochrome b | 0.5 | TTGGTGCTAGAGATTATTCTGTTCCT | Integrated DNA Technologies |
|  | *Pf*cytb_m_R2 | cytochrome b | 0.5 | GGAGCTGTAATCATAATGTGTTCGTC | Integrated DNA Technologies |
| *P. malariae* | Mal-F | RNA 18s | 0.5 | CCGACTAGGTGTTGGATGATAGAGTAAA | Integrated DNA Technologies |
|  | Plasmo2-R | RNA 18s | 0.5 | AACCCAAAGACTTTGATTTCTCATAA | Integrated DNA Technologies |
| *P. ovale* | O_FWD | DHFR | 0.5 | GGKCTTGGTGTTCCCTTCA | Integrated DNA Technologies |
|  | O_Rev | DHFR | 0.5 | TGTGRGCATTTCCTAAAACG | Integrated DNA Technologies |
